# One-stop-shop CT arthrography of the wrist without subject repositioning by means of gantry-free cone-beam CT

**DOI:** 10.1038/s41598-022-18395-2

**Published:** 2022-08-24

**Authors:** Karsten Sebastian Luetkens, Jan-Peter Grunz, Mila Marie Paul, Henner Huflage, Nora Conrads, Theresa Sophie Patzer, Philipp Gruschwitz, Süleyman Ergün, Thorsten Alexander Bley, Andreas Steven Kunz

**Affiliations:** 1 Department of Diagnostic and Interventional Radiology, University Hospital Würzburg, Oberdürrbacher Straße 6, 97080 Würzburg, Germany; 2 Department of Orthopedic Trauma, Hand, Plastic and Reconstructive Surgery, University Hospital Würzburg, Oberdürrbacher Straße 6, 97080 Würzburg, Germany; 3 Institute of Anatomy and Cell Biology, University of Würzburg, Koellikerstr. 6, 97070 Würzburg, Germany

**Keywords:** Medical research, Preclinical research

## Abstract

Modern cone-beam CT systems are capable of ultra-high-resolution 3D imaging in addition to conventional radiography and fluoroscopy. The combination of various imaging functions in a multi-use setup is particularly appealing for musculoskeletal interventions, such as CBCT arthrography (CBCTA). With this study, we aimed to investigate the feasibility of CBCTA of the wrist in a “one-stop-shop” approach with a gantry-free twin robotic scanner that does not require repositioning of subjects. Additionally, the image quality of CBCTA was compared to subsequent arthrograms on a high-end multidetector CT (MDCTA). Fourteen cadaveric wrists received CBCTA with four acquisition protocols. Specimens were then transferred to the CT suite for additional MDCTA. Dose indices ranged between 14.3 mGy (120 kVp/100 effective mAs; full-dose) and 1.0 mGy (70 kVp/41 effective mAs; ultra-low-dose) for MDCTA and between 17.4 mGy (80 kVp/2.5 mAs per pulse; full-dose) and 1.2 mGy (60 kVp/0.5 mAs per pulse; ultra-low-dose) for CBCTA. Subjective image quality assessment for bone, cartilage and ligamentous tissue was performed by seven radiologists. The interrater reliability was assessed by calculation of the intraclass correlation coefficient (ICC) based on a two-way random effects model. Overall image quality of most CBCTA was deemed suitable for diagnostic use in contrast to a considerable amount of non-diagnostic MDCTA examinations (38.8%). The depiction of bone, cartilage and ligaments in MDCTA with any form of dose reduction was inferior to any CBCTA scan with at least 0.6 mAs per pulse (all p < 0.001). Full-dose MDCTA and low-dose CBCTA were of equal quality for bone tissue visualization (p = 0.326), whereas CBCTA allowed for better depiction of ligaments and cartilage (both p < 0.001), despite merely one third of radiation exposure (MDCTA–14.3 mGy vs. CBCTA–4.5 mGy). Moderate to good interrater reliability was ascertained for the assessment all tissues (ICC 0.689–0.756). Overall median examination time for CBCTA was 5.4 min (4.8–7.2 min). This work demonstrates that substantial dose reduction can be achieved in CT arthrography of the wrist while maintaining diagnostic image quality by employing the cone-beam CT mode of a twin robotic X-ray system. The ability of the multi-use X-ray system to switch between fluoroscopy mode and 3D imaging allows for “one-stop-shop” CBCTA in minimal examination time without the need for repositioning.

## Introduction

While wrist pain represents a common medical condition^1^, causes can range from fractures to cartilage injuries to discontinuity of ligamentous structures. Lesions of the intrinsic carpal ligaments and the triangular fibrocartilage complex (TFCC) are often concomitant findings in both traumatic or degenerative conditions of the wrist. Tears of the scapholunate ligament are present in 10% of distal radius fractures^2^, while injuries of the lunotriquetral ligament are more infrequent^3,4^. Lesions of these fine stabilizers correspond with changes in the biomechanics of the wrist and eventually invoke osteoarthritis and chronic pain^5,6^. While degenerative lesions of the TFCC are usually asymptomatic, traumatic injuries commonly result in ulnar-sided wrist pain^7^. Due to overlapping symptoms and the complex anatomy of the wrist, identifying subtle soft tissue injuries remains a challenging task in clinical examinations of patients with wrist fractures. Furthermore, neither first-line radiography nor standard unenhanced CT are capable of directly visualizing ligamentous or cartilaginous lesions, albeit the latter provides viable information on fracture morphology and fragment displacement^8^. To prevent late complications like carpal instability, however, more sophisticated imaging techniques can be required with certain fracture patterns. Plain and contrast-enhanced MRI occasionally struggle with discreet lesions like isolated ruptures of the foveal attachment of the TFCC, which represents the main stabilizer of the DRUJ^9^, or chondral lesions of small articular surfaces^10–12^. Hence, regardless of drawbacks such as lengthy examinations and uncomfortable positioning^13–15^, MR arthrography is often regarded as the method of choice for imaging the intrinsic ligaments and the TFCC^16–18^. However, previous studies have reported comparable sensitivity and specificity levels for CT arthrography^18–20^. While multidetector CT arthrography (MDCTA) represents the most common approach to this technique, the emergence of dedicated extremity scanners with flat-panel detectors and cone-shaped beam geometry has opened up a new option in the form of cone-beam CT arthrography (CBCTA)^21^. Superior results have already been reported for plain CBCT in comparison to MDCT of the appendicular skeleton^22^. More importantly, though, new types of scanner architecture, such as the twin robotic design of the X-ray system employed in this study, offer a wider range of functionality, e.g. by combining fluoroscopy with ultra-high-resolution 3D imaging in the same position.

Due to the current lack of data on that topic, this experimental study was performed to investigate the feasibility of “one-stop-shop” CBCTA in cadaveric specimens. Using various scan protocols to define the best possible trade-off between image quality and radiation dose, we hypothesized that CBCTA would provide low examination times while yielding superior ratings over standard MDCTA in a multi-observer analysis.

## Material and methods

### Cadaveric phantoms

**Figure 1:**
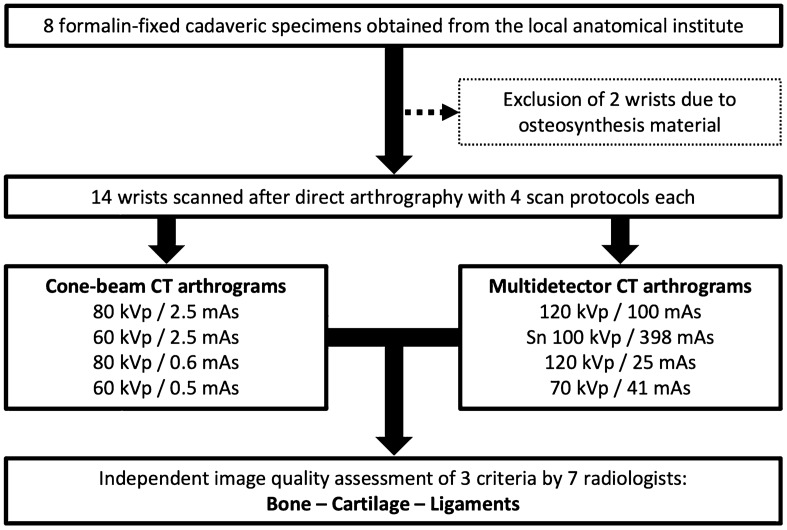
Flow chart illustrating the study design.

Eight formalin-fixed cadaveric specimens were provided by the anatomical institute of the local university. The body donors had provided written informed consent to donate their remains posthumously for study and research purposes. All experimental protocols were approved and further informed consent was waived by the Institutional Review Board of the University of Würzburg, Germany (protocol number: 2020050601). Additional written informed consent was waived by the ethics committee. Figure 1 illustrates the study design. Of the total of 16 wrists available, two wrists had to be excluded from the study due to implanted osteosynthesis material in the distal radius. Therefore, the final study sample consisted of 14 cadaveric wrists. The datasets generated and/or analysed during the current study are not publicly available but are available from the corresponding author on reasonable request. Due to the nature of this research, participants of this study did not agree for their data to be shared in a public repository. All methods were carried out in accordance with relevant guidelines and regulations.

### Arthrography

**Figure 2:**
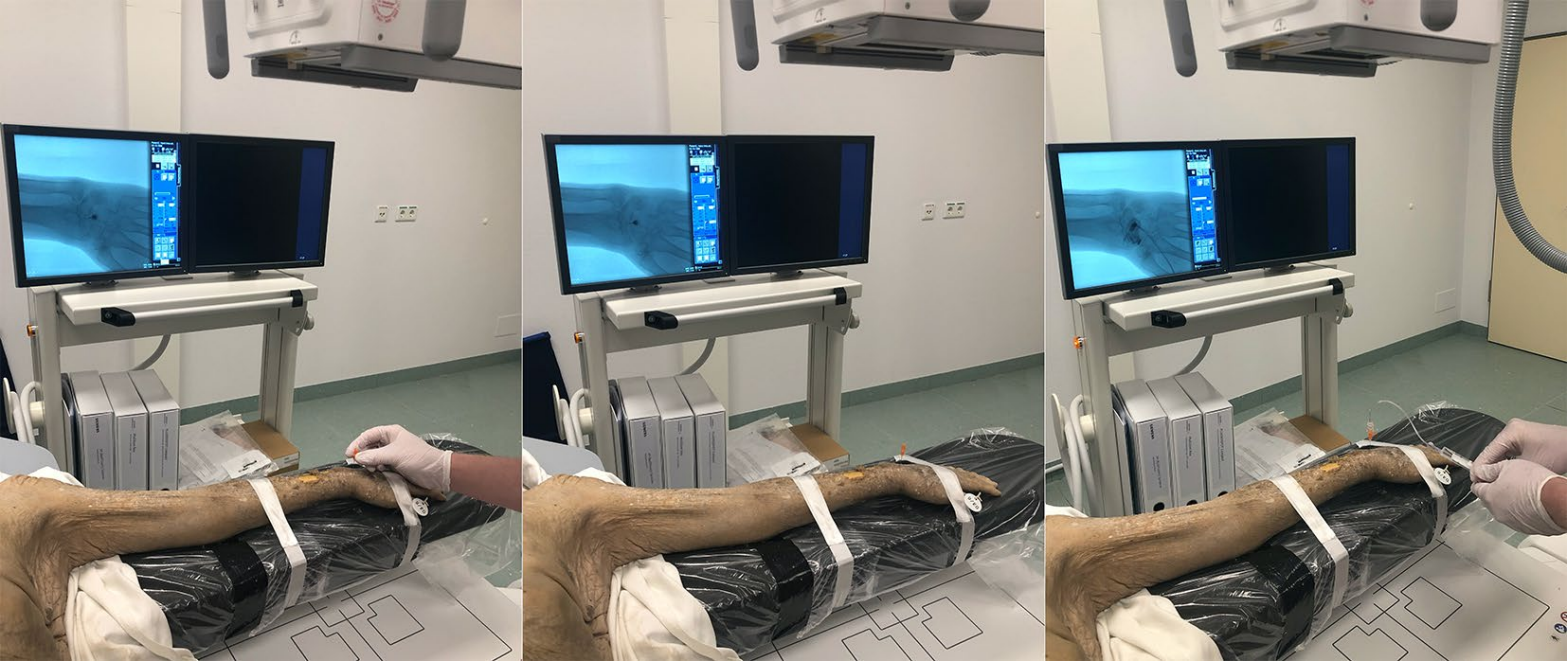
Fluoroscopy-guided arthrography of the midcarpal joint of a cadaveric specimen using the tableside scan trajectory of the twin robotic x-ray system. The specimen remained in the same position for the ultra-high-resolution cone-beam CT scans, allowing for a very short examination time.

Each arthrography was performed by a board-certified radiologist with 7 years of experience in musculoskeletal imaging employing the fluoroscopy mode of the multi-use X-ray system (Multitom Rax, Siemens Healthineers, Erlangen, Germany) for guidance. Specimens were brought in a supine position with the respective arm abducted by approximately 90 degrees (Fig. 2). For the procedure of articular contrast injection, the acquisition of fluoroscopy images was controlled by the scanner’s multifunctional footswitch. A 25 gauge needle (BD Eclipse™ Smartslip, Becton, Dickinson and Company, Franklin Lakes, New Jersey, United States of America) was utilized to puncture the articular capsule. To mimic the examination of real-world patients, a mixture of iodine contrast medium with a concentration of 300 mg per ml (Imeron 300^®^, Bracco S.p.A., Milan, Italy) and anaesthetic with a concentration of 10 mg per ml (Mecain^®^, Puren Pharma GmbH & Co. KG, Munich, Germany) was injected. For assessment of the intrinsic carpal ligaments, the midcarpal joint was punctured first between the lunate, triquetral, capitate and hamate bone, followed by the radiocarpal joint adjacent to the proximal scaphoid pole. Injected volumes ranged between 2–4 ml (midcarpal joint) and 1.5–3 ml (radiocarpal joint).

### Scanner and scan protocols

After completing the arthrography procedure, all CBCT scans were acquired without further delay and without repositioning of the cadaveric specimens. The gantry-free multifunctional X-ray system consists of two ceiling rails with mounted robotic arms, which simultaneously and synchronously navigate a quadratic flat-panel detector and the X-ray tube in an asymmetric source-to-image distance (1150 mm) and a sweep angle of 200° around the isocenter in the 3D CBCT mode. The resulting total scan time is 14 s. With the current software version VF11 (Siemens Healthineers), the unbinned readout of the detector in ultra-high-resolution mode results in a 1440 × 1440 matrix of pixels with an effective size of 149 μm.

The four CBCT scan protocols comprised of tube voltages between 60 and 80 kVp and current–time products between 0.5 and 2.5 mAs. The scanner-immanent prefiltration was 0.3 mm of copper. Time intervals were recorded for each step of the arthrogram and 3D imaging process. After completing all CBCT scans, the specimens were immediately transferred to a high-end, gantry-based MDCT scanner (Somatom Force, Siemens Healthineers) and brought in prone position with the wrist elevated above the head (“superman” position). The four MDCT scan protocols varied between 70 and 120 kVp tube voltage and 25–398 mAs current–time product. One MDCT protocol employed tin prefiltration (0.6 mm).

In total, eight different scan protocols were defined for the purpose of this study. The individual detailed combinations are summarized in Table 1. The radiation dose in MDCT scans was determined according to the dose-length product and the volume CT dose indices for a 16 cm phantom (CDTI_vol_). For comparison, CTDI_vol_ equivalents were calculated for all CBCT scans by multiplying dose-area-products by a linear scaling factor that was evaluated in advance for each scan protocol. Dose-length-product measurements were based on a conventional polymethylmethacrylate dosimetry phantom (IEC 60601-2-44:2009) with a diameter of 16 cm. Standard weighting factors were applied to obtain volume dose-length-products, which then were divided by the field of view in z-axis, i.e. by the beam width. The volume computed tomography dose indices were then divided by the dose-area-products of the automatic dose report, resulting in the required scaling factor.

**Table 1:** Scan protocols.

Parameters	Multidetector CT arthrography				Cone-beam CT arthrography			
Voltage [kVp]	120	100	120	70	80	60	80	60
Current-time product [mAs]	100	398	25	41	2.5	2.5	0.6	0.5
Filter [mm]	–	Sn 0.6	–	–	Cu 0.3	Cu 0.3	Cu 0.3	Cu 0.3
CTDI_vol_ [mGy]	14.3	3.7	3.4	1.0	17.4	9.5	4.5	1.2

Scan protocols and radiation dose for multidetector and cone-beam CT arthrograms.

*Cu* standard copper prefiltration, *Sn* tin prefiltration, *CTDI*_*vol*_ volume computed tomography dose index for 16 cm phantom.

### Image reconstruction parameters

Based on the clinical standard procedure for post-processing, all scanner-side raw data image reconstructions were performed with a high-resolution bone kernel (Ur77; Siemens Healthineers). Multiplanar reconstructions in axial, coronal and sagittal planes were conducted for all scans utilizing a dedicated 3D processing software (syngo.via View&GO and syngo.via, both Siemens Healthineers) in 1.0 mm slice thickness with an increment of 0.5 mm, a field of view of 80 mm, and an image matrix of 1024 × 1024 pixels. For identical image presentation, reconstruction processes were identical for each scanner type and scan protocol. Basic values for window width and level were selected at 3000 and 1000 Hounsfield units (HU) for optimal bone tissue depiction. However, observers were permitted to modify window settings at will for reading.

### Image evaluation

Images were evaluated by seven radiologists independently on a certified diagnostic monitor (RadiForce RX660, EIZO, Hakusan, Japan) with a standard picture archiving and communication software (Merlin, Phönix-PACS, Freiburg, Germany). The radiologists’ level of experience in musculoskeletal imaging ranged between two and eight years. Without having been provided any further information regarding image acquisition, readers were first tasked to evaluate whether images were suitable for diagnostic use. Subsequently, the observers were requested to individually rate the image quality for bone, cartilage and the intrinsic ligaments using a seven-point scale (1 = very poor; 2 = poor; 3 = fair; 4 = satisfactory; 5 = good; 6 = very good; 7 = excellent). Image noise was measured in normed regions of interest to assess image quality quantitatively.

### Statistical analysis

Statistical software (SPSS, IBM, Armonk, USA) was employed for all analyses. Kolmogorov–Smirnov tests were performed to assess normal distribution in cardinal variables. Ordinal variables are presented as absolute and relative frequencies with median values and interquartile ranges (IQR, 25–75%). Friedman tests and Bonferroni-corrected pairwise post-hoc analyses were conducted to compare the mean rank distribution in paired non-parametric variables. Null hypotheses were rejected and statistical significance assumed if computed p values were ≤ 0.05. To test for interrater agreement, the intraclass correlation coefficient (ICC) was calculated for absolute agreement of single measures in a two-way random effects model. ICC scores were interpreted following Koo and Li: < 0.50: poor; 0.50–0.75: moderate; 0.75–0.90: good; > 0.90: excellent reliability^23^.

## Results

**Figure 3:**
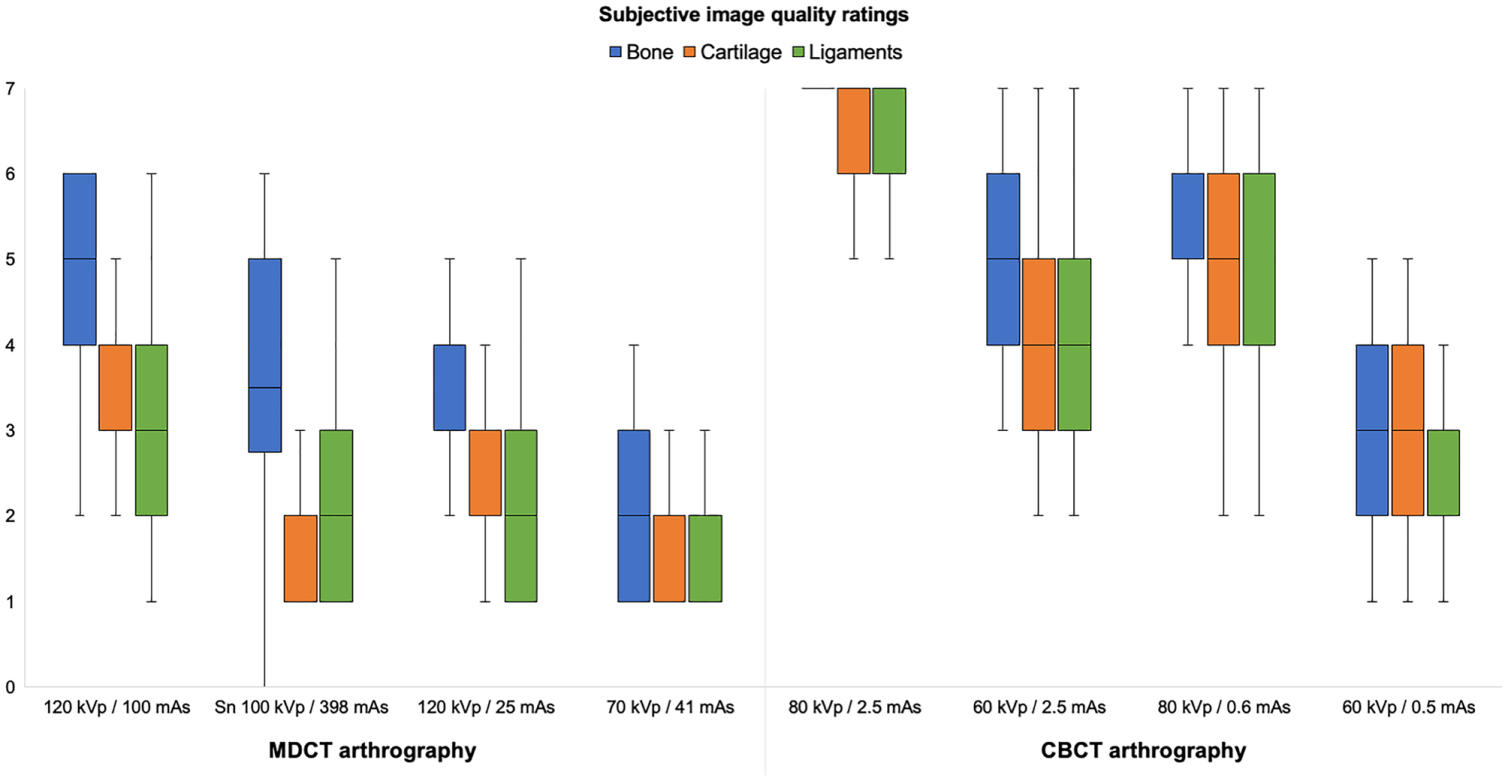
Box and whisker plots depict the subjective image quality ratings regarding bone, cartilage, and ligaments by seven radiologists for multidetector CT (MDCT) and cone-beam CT (CBCT) arthrography.

**Figure 4:**
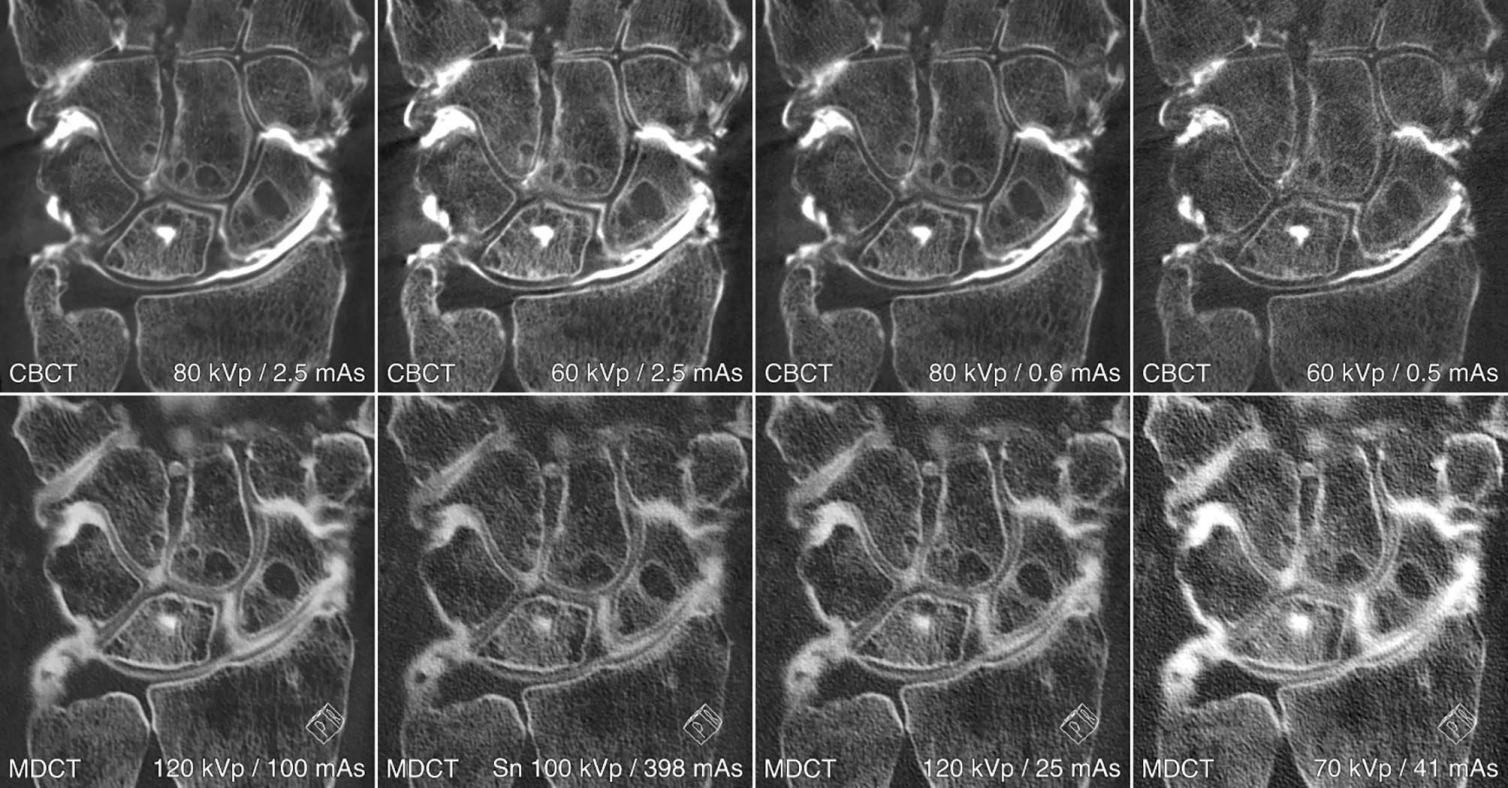
Coronal view of various scan protocols (in descending order of radiation dose) depicts a scapholunate ligament tear. Visualization of the tear’s location in the scaphoid portion of the ligament is superior in cone-beam CT scan (CBCT; upper row). Despite being acquired 30 minutes later, the blurriness of multidetector CT images (MDCT; lower row) partially offsets the advantage of increased articular distension for cartilage assessment, for example in the radiocarpal compartment.

**Figure 5:**
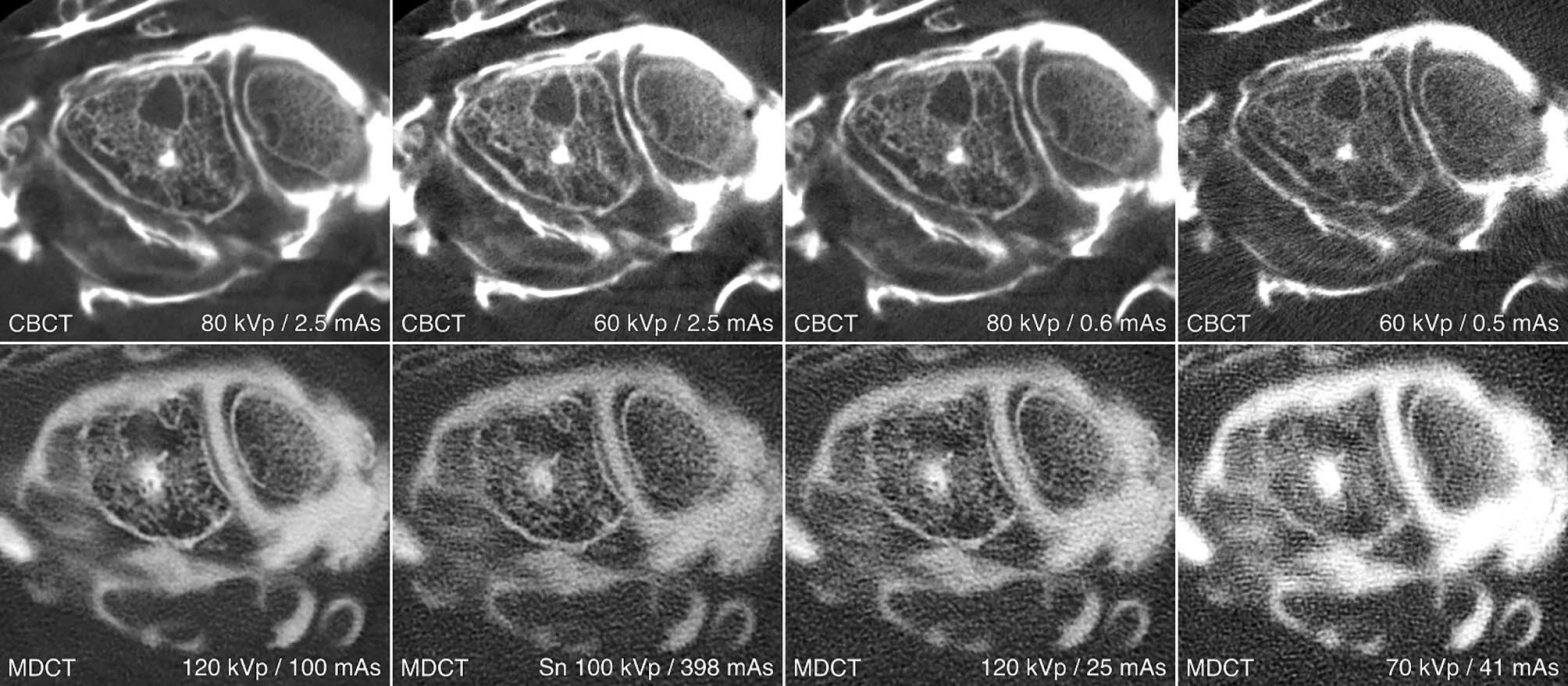
Axial views of the same cadaveric specimen depict the torn dorsal portion of the scapholunate ligament, which functions as the primary stabilizer of the proximal carpal row. While increased articular distension over time was helpful for ligament analysis in multidetector CT (MDCT; lower row), the detailed visualization of bone microarchitecture in cone-beam CT (CBCT; upper row) allowed for superior depiction of a ganglion cyst within the lunate bone.

Depending on the acquisition parameters of the respective 3D scan protocol, CTDI_vol_ in this study ranged between 1.0 and 17.4 mGy (Table 1). The overall image quality of most CBCT arthrograms was considered suitable for use in clinical routine (386/392 ratings, 98.5% examinations in diagnostic quality), whereas one third of MDCTA scans was deemed insufficient for patient imaging (244/392, 62.2% examinations in diagnostic quality). Detailed subjective image quality ratings are summarized in Table 2. The best image quality in each category was achieved with the full-dose CBCTA protocol (80 kVp, 2.5 mAs per pulse), which also induced the highest radiation dose (17.4 mGy) (Fig. 3). While no difference was ascertained between full-dose MDCTA (120 kVp, 100 mAs) and low-dose CBCTA (80 kVp, 0.6 mAs) for bone tissue visualization (p = 0.326), CBCTA scans allowed for better depiction of ligaments and cartilage (both p < 0.001), despite being associated with just one third of radiation exposure (MDCTA—14.3 mGy vs. CBCTA—4.5 mGy). Figure 4 illustrates the superior image quality of CBCTA in a cadaveric specimen with scapholunate ligament tear. Irrespective of the tissue assessed, no significant difference was recorded between CBCTA scans with 80 kVp/0.6 mAs and 60 kVp/2.5 mAs (all p ≥ 0.618), although the latter protocol resulted in twice the radiation dose (80 kVp–4.5 mGy vs. 60 kVp–9.5 mGy). Tin-filtered MDCTA with 100 kVp were considered equal to low-dose MDCTA studies with reduced tube current or potential (all p ≥ 0.204). Notably, however, all MDCTA protocols with any form of dose reduction were deemed inferior to CBCTA scans with at least 0.6 mAs per pulse (bone/cartilage/ligaments: all p < 0.001). Between ultra-low-dose examinations, CBCTA with 60 kVp/0.5 mAs were rated better than MDCTA with 70 kVp/41 mAs for each of the three categories (all p < 0.001). The superior visualization of bone microarchitecture and alterations thereof in CBCTA is exemplified in Fig. 5. Detailed results of pairwise comparison between the scan protocols on either scanner are shown in Table 3. Interrater reliability was moderate—good for bone tissue (ICC 0.690 [95% confidence interval 0.555–0.787; p < 0.001]), cartilage (0.756 [0.672–0.823; p < 0.001]) and ligament assessment (0.689 [0.586–0.772; p < 0.001]). Image noise was lowest and highest in CBCTA scans with 80 kVp/2.5 mAs and 60 kVp/0.5 mAs, respectively. For MDCTA, lowest and highest noise was measured in examinations with 120 kVp/100 mAs and 70 kVp/41 mAs. Detailed noise levels are presented in Table 4.

**Table 2:** Image quality ratings.

Image quality	Multidetector CT arthrography				Cone-beam CT arthrography			
kVp/mAs	120/100	Sn 100/398	120/25	70/41	80/2.5	60/2.5	80/0.6	60/0.5
Bone	5 (4–5)	4 (3–5)	3.5 (3–4)	2 (1–2)	7 (7–7)	5 (4–6)	6 (5–6)	4 (3–4)
Cartilage	3 (3–4)	2 (1–2)	2 (2–3)	1 (1–2)	6 (5–7)	4 (3–5)	4 (4–6)	3 (2–4)
Ligaments	3 (3–4)	2 (1–2)	2 (1–3)	1 (1–2)	6 (5–7)	3 (3–5)	4 (3–5)	3 (2–3)
Percentage of diagnostic examinations	93.9	43.9	77.6	33.7	100.0	100.0	100.0	93.9

Pooled image quality ratings of seven observers for bilateral wrist arthrograms in eight cadaveric specimens. Results are presented as median values with interquartile ranges in parentheses.

*kVp* kilovoltage peak, *mAs* milliampere-seconds, *Sn* tin prefiltration.

**Table 3:** Subjective image quality comparison.

Bone/cartilage/ligaments		Multidetector CT arthrography				Cone-beam CT arthrography			
	kVp/mAs	120/100	Sn 100/398	120/25	70/41	80/2.5	60/2.5	80/0.6	60/0.5
Multidetector CT arthrography	120/100	x	+/+/+	+/+/+	+/+/+	−/−/−	=/=/=	=/−/−	+/=/=
	Sn 100/398	−/−/−	x	=/=/=	+/=/=	−/−/−	−/−/−	−/−/−	=/−/−
	120/25	−/−/−	=/=/=	x	+/+/+	−/−/−	−/−/−	−/−/−	=/=/=
	70/41	−/−/−	−/=/=	−/−/−	x	−/−/−	−/−/−	−/−/−	−/−/−
Cone-beam CT arthrography	80/2.5	+/+/+	+/+/+	+/+/+	+/+/+	x	+/+/+	+/+/+	+/+/+
	60/2.5	=/=/=	+/+/+	+/+/+	+/+/+	−/−/−	x	=/=/=	+/+/+
	80/0.6	=/+/+	+/+/+	+/+/+	+/+/+	−/−/−	=/=/=	x	+/+/+
	60/0.5	−/=/=	=/+/+	=/=/=	+/+/+	−/−/−	−/−/−	−/−/−	x

Mean image quality rank compared between scan protocols for bone/cartilage/ligaments with pairwise post-hoc analyses.

*kVp* kilovoltage peak, *mAs* milliampere-seconds, *Sn* tin prefiltration.

p values of pairwise post-hoc tests were Bonferroni-corrected for multiple comparisons.

“***+***”: superior image quality; “−”: inferior image quality; “***=***”: no statistically significant difference in image quality.

**Table 4:** Quantitative image quality comparison.

	Multidetector CT arthrography				Cone-beam CT arthrography			
kVp/mAs	120/100	Sn 100/398	120/25	70/41	80/2.5	60/2.5	80/0.6	60/0.5
Median noise	128.0	210.1	218.3	371.9	98.2	167.5	156.4	1040.6
Interquartile range	111.2–143.3	182.0–249.3	195.8–267.4	347.1–408.0	91.6–102.0	151.1–184.8	145.0–171.1	885.2–1105.6

Image noise quantification for multidetector CT (MDCT) and cone-beam CT (CBCT) arthrography.

The median time required for the fluoroscopy-guided two-compartment wrist arthrography was 2.8 min (IQR 2.0–5.0 min). Irrespective of scan protocol, the median time interval between the final contrast injection (into the radiocarpal joint) and completion of the automated scanner-side image reconstruction in orthogonal planes was 2.4 min (IQR 2.2–2.4 min). Time requirements for each step of gantry-free CBCTA are summarized in Table 5.

**Table 5:** Examination time.

Time [s]	Arthrography	Acquisition + reconstruction	Overall
Median (IQR)	165.9 (117.3–297.1)	145.2 (132.0–146.4)	320.9 (285.5–433.6)
Mean ± SD	253.4 ± 200.0	149.6 ± 33.1	403.0 ± 198.2

Time requirements for fluoroscopy-guided three-compartment arthrography with subsequent ultra-high-resolution cone-beam CT using the gantry-free twin robotic X-ray system.

Time intervals were recorded for each step of the arthrogram and 3D imaging process by a dedicated timekeeper.

*IQR* interquartile range, *SD* standard deviation.

## Discussion

In this experimental study, we demonstrated the feasibility of combining fluoroscopy-guided wrist arthrography and ultra-high-resolution CBCT in a “one-stop-shop” approach using a multipurpose, twin robotic X-ray system. Comparing the image quality with various dose levels to the performance of a high-end MDCT scanner, our findings suggest significant dose reduction potential for ultra-high-resolution CBCTA. Particularly, the considerable amount of non-diagnostic MDCTA examinations has to be mentioned when any form of dose reduction was employed. In contrast, any CBCTA protocol with at least 0.6 mAs per pulse provided diagnostic image quality in every scan. One of the major drawbacks of CT arthrography in comparison to MR arthrography, i.e. the radiation exposure, can thus be minimized. The findings in this study correlate with a recent meta-analysis by Nardi et al. for plain CBCT, which demonstrated significant dose reduction potential in comparison to MDCT for imaging tasks concerning the appendicular skeleton^24^. The sustained subjective and quantitative image quality in ultra-low-dose CBCTA may be mostly interrelated with the superior spatial resolution, which is derived from the combination of an asymmetric acquisition geometry and unbinned readout of the flat-panel detector with an isotropic pixel size of 149 µm. For comparison, the employed MDCT systems’ ultra-high-resolution scan mode allows for 300 µm in the axial plane and 400 µm in z-direction (with a gantry rotation time of 1 s).

In literature, the diagnostic value of wrist MDCTA is described to be equivalent or even better than MRI or MR arthrography for lesions of the cartilage, intrinsic carpal ligaments and TFCC^18,20^. Especially in small joints with thin hyaline cartilage, MRI, which is often seen as the imaging technique of choice in clinical settings, struggles regarding resolution and depiction of discreet chondral injuries^11,25,26^. Although the sensitivity and specificity for these lesions could be improved by direct MR arthrography up to 84–96%^11,27^, CBCTA of the wrist should be considered as an alternative imaging technique with higher spatial resolution and significantly shorter acquisition time. This combination may be beneficial not only for patients with contraindications for MRI but also for patients unable to maintain the required scan position (“superman” position) over a longer time. In addition, CT arthrography is advantageous regarding detailed evaluation of the subchondral surface in case of cystic or sclerotic alterations, while MR arthrography maintains the advantage of superior assessment of bone marrow abnormalities^10,28^.

Finally, the gantry-free cone-beam CT with two robotic arms used in this study allows for a “one-stop-shop” approach to wrist arthrography with a median time interval of 2.4 min between the final contrast injection and completion of the scanner-side image reconstruction in orthogonal planes. This is possible because patient repositioning or even relocation between fluoroscopy and CT suite is not necessary. The subject remains in a comfortable scan position with the arm abducted at 90°, eliminating delays and reducing logistical challenges. Although studies have reported that a significant amount of contrast medium remains in the joint space for up to 120 min in delayed CT arthrography^29^, a short time interval between injection and imaging is recommended to receive optimal contrast conditions. Further studies are required to analyze the impact of the time aspect in clinical settings.

### Limitations

Regarding this study, several limitations are to be mentioned. Receiving the specimens without further information on body donor age, time of fixation and bone density, preexisting osteopenia, as well as the bone demineralization in formalin could have a negative influence on the subjective image analysis^30,31^. Also, acquiring the MDCTA scans in a median time interval of 30 min after injection may have led to accumulation of intraarticular contrast volume in comparison to CBCTA examinations, resulting in superior joint distension. Also, by examining cadaveric wrists, the impact of potential motion artefacts and off-center positioning with an overall scan time of 12 s in CBCT were not evaluated, each of which could conceivably impair the image quality regardless of the benefits of comfortable table-side positioning. As MDCT systems generally require shorter scan times^18^, it remains unclear whether the comfortable stance offsets the disadvantages inherent to longer scan times in CBCT. At last, despite being blinded to image acquisition parameters, the observers may have become more acquainted with imaging characteristics throughout their reading sessions.

## Conclusion

With its twin robotic arms, the multi-use X-ray system enables fluoroscopy-guided wrist arthrography and subsequent ultra-high-resolution cone-beam CT in a “one-stop-shop” approach without subject repositioning. In addition to time savings, the combined procedure of cone-beam CT arthrography provides superior image quality compared to standard MDCT arthrograms and holds potential for significant radiation dose reduction.

## Data Availability

The datasets used and/or analysed during the current study available from the corresponding author on reasonable request.

## Abbreviations

CBCTA: Cone-beam computed tomography arthrography
CTDI_vol_: Volume computed tomography dose index (for 16 cm phantom)
MDCTA: Multidetector computed tomography arthrography
TFCC: Triangular fibrocartilage complex
